# Complete genome sequence of *Bacillus cereus* NBNZ-2162, isolated from citrus rhizosphere soil

**DOI:** 10.1128/mra.00696-24

**Published:** 2024-10-21

**Authors:** Wei Chen, Ling Chen, Xiyu Cheng, Xiaoyan Liu, Lei Zhu

**Affiliations:** 1 National Biopesticide Engineering Technology Research Centre, Hubei Biopesticide Engineering Research Centre, Hubei Academy of Agricultural Sciences, Biopesticide Branch of Hubei Innovation Centre of Agricultural Science and Technology, Wuhan, China; 2 School of Life and Health Science, Hubei University of Technology, Wuhan, China; University of Strathclyde, Glasgow, United Kingdom

**Keywords:** *Bacillus cereus*, citrus, rhizosphere soil, genomes

## Abstract

Plant rhizosphere microorganisms are an important source for discovering beneficial microorganisms in crops. Here we presented the complete genome of *Bacillus cereus* NBNZ-2162 isolated from citrus rhizosphere soil, of which the total length is 5,395,922 bp and possesses 5,368 CDS.

## ANNOUNCEMENT

Plant rhizosphere microorganisms are an important source for discovering and creating microbial agents for crop disease prevention and growth promotion, especially in the field of *Bacillus* preparations, where there have been multiple successful cases (1, 2). Strain NBNZ-2162 was isolated in June 2021 from citrus rhizosphere soil that tightly adhered to the root system in Yangxin, Hubei Province, China. One gram of soil samples was serially diluted with sterile water and was spread on lysogeny broth (LB) medium plates. After incubation overnight at 30°C, a single colony was purified by three rounds of scribing on LB plates. To characterize the strain, the 16S rRNA gene sequence was obtained by PCR amplification with the universal primers 27F and 1492R (3). Phylogenetic analysis was performed using EZBioCloud database (4). The comparison results showed the greatest similarity to the *B. cereus* type strain ATCC 14579(T) (99.93% identity, GenBank accession number AE016877). Here, we report the complete genome sequence of *B. cereus* NBNZ-2162.

The purified colony was inoculated into LB medium and cultivated at 30°C to the middle logarithmic growth stage. Genomic DNA was prepared using the Blood & Cell Culture DNA Kits (QIAGEN, Germany) according to the manufacturer’s instructions. DNA’s concentration was detected by NanoDrop One UV-Vis spectrophotometer (Thermo Fisher Scientific, USA) and Qubit 4.0 Fluorometer (Invitrogen, USA). Sample integrity and purity were detected by 0.75% agarose gel electrophoresis. The long- and short-read sequencings were performed from the same DNA preparation. For long-read sequencing, size-select (>10 kb) were performed using the PippinHT system (Sage Science, USA). The DNA library was prepared using a ligation sequencing kit (SQK-LSK-109; Oxford Nanopore Technologies, Ltd. [ONT], Oxford, UK) without DNA shearing and was sequenced with a Nanopore PromethION sequencer instrument (ONT) on an R9.4.1 flow cell (FLO-MIN106). Base-calling, barcode segmentation and adapter sequences removement for the raw sequences were performed using Guppy v.5.0.16 (5). 145,727 reads (1,090 Mb) were generated with an average length of 7,477 bp, and N50 was 12,586 bp. For short-reads sequencing, genomic DNA was randomly fragmented by Covaris LE220 focused ultrasonicator (USA), and the fragmented DNA was selected by AMPure XP magnetic beads (Beckman, Germany) to an average size of 200–400 bp. Paired-end DNA library was prepared following the MGIEasy FS PCR Free DNA library preparation set (MGI Tech Co., Ltd.; catalog number 1000013455). Sequencing was performed on a DNBSEQ-T7 instrument (MGI, China), using 150 nucleotide length reads. Raw data were processed using the FASTQ preprocessing program fastp v.0.23.2 (6) for the purpose of trimming adapters and low-quality data, yielding 7,584,032 short reads with a total length of 1,127,517,036  bp.

The *B. cereus* NBNZ-2162 genome was assembled into two circular replicons, with one circular chromosome (5,163,372 bp, GC content of 35.33%) and one circular plasmid (232,550 bp, GC content of 32.22%) using Unicycler v.0.5.0 (7). The average coverage is 395.92. The total genome length is 5,395,922 bp, containing 5,368 CDS, 106 tRNA genes, and 42 rRNA genes, as determined by NCBI PGAP v.6.7 (8). In this study, default parameters were used for all software.

## Data Availability

The 16S rRNA gene has been deposited at GenBank under the accession number PP917503.1. The whole genome is available in GenBank under accession numbers CP158496 and CP158497. The raw sequencing data were deposited in the Sequence Read Archive (SRA) database under accession numbers SRR29430136 (MGI) and SRR29430137 (ONT).
